# Meta-analysis of thyroidectomy with ultrasonic dissector versus conventional clamp and tie

**DOI:** 10.1186/1477-7819-8-112

**Published:** 2010-12-23

**Authors:** Roberto Cirocchi, Fabio D'Ajello, Stefano Trastulli, Alberto Santoro, Giorgio Di Rocco, Domenico Vendettuoli, Fabio Rondelli, Domenico Giannotti, Alessandro Sanguinetti, Liliana Minelli, Adriano Redler, Antonio Basoli, Nicola Avenia

**Affiliations:** 1 General and Emergency Surgical Unit. Department of Surgical Sciences, Radiology and Dentistry. University of Perugia, Perugia, Italy; 2 Endocrine Surgical Unit. Department of Surgical Sciences, Radiology and Dentistry. University of Perugia, Perugia, Italy; 3 Department of Surgical Sciences. Sapienza University of Rome, Rome, Italy; 4 Public Health Department. University of Perugia, Perugia, Italy; 5 Department Paride Stefanini. Sapienza University of Rome, Rome, Italy

## Abstract

**Background:**

We conducted a systematic review to evaluate the role of Ultrasonic dissector (UAS) versus conventional clamp and tie in thyroidectomy.

**Materials and methods:**

We searched for all published RCT in into electronic databases. To be included in the analysis, the studies had to compare thyroidectomy with UAS versus conventional vessel ligation and tight (conventional technique = CT). The following outcomes were used to compare the total thyroidectomy group with UAS versus CT group: operative duration, operative blood loss, overall drainage volume during the first 24 hours, transiet laryngeal nerve palsy, permanent laryngeal nerve palsy, transiet hypocalcaemia and permanent hypocalcaemia.

**Results:**

There are currently 7 RCT on this issue to compare thyroidectomy with UAS versus CT. From the analysis of these studies it was possible to confront 608 cases: 303 undergoing to thyroidectomy with UAS versus 305 that were treated with CT. Actually, it was shown a relevant advantage of cost-effectiveness in patients treated with UAS; there is a statistically significant reduction of the operative duration (weighted mean difference [WMD], -18.74 minutes; 95% confidence interval [CI], (-26.97 to -10.52 minutes) (P = 0.00001), intraoperative blood loss (WMD, -60.10 mL; 95% CI, -117.04 to 3.16 mL) (P = 0.04) and overall drainage volume (WMD, -35.30 mL; 95% CI, -49.24 to 21.36 mL) (P = 0.00001) in the patients underwent thyroidectomy with UAS. Although the analysis showed that the patients who were treated with USA presented more favourable results in incidence of post-operative complications (transient laryngeal nerve palsy: P = 0.11; permanent laryngeal nerve palsy: not estimable; transient hypocalcaemia: P = 0.24; permanent hypocalcaemia: P = 0.45), these data didn't present statistical relevance.

**Conclusion:**

This meta-analysis shown a relevant advantage only in terms of cost-effectiveness in patients treated with UAS; it is subsequent to statistically significant reduction of operation duration, intraoperative blood loss and of overall drainage volume during the first 24 hours. Although the analysis showed that the patients who were treated with UAS presented more favourable results in incidence of post-operative complications (transiet laryngeal nerve palsy; transiet hypocalcaemia and permanent hypocalcaemia), these data didn't present statistical relevance.

## Introduction

The basis for the thyroid surgery was founded by Theodor Billroth and Theodor Kocher, between 1873 and 1893, that standardized and precise anatomical dissection with preliminary ligation of the two principal arteries of the gland on each side, followed by excision of the gland[1].

During the last ten years MIVAT (Minimally invasive video assisted throidectomy) was standardized by Miccoli in 1999[2], and various devices were introduced in order to do a safe section and haemostasis of thyroidal vessels (LigaSure and Ultrasonic dissector)[3]. The Ultracision dissector (UAS) (First generation "Harmonic scalpel"; Johnson & Johnson^®^) is a device that uses vibration at 55,5 KHz simultaneously cut and coagulate tissue. The UAS works at lower temperature (ranging from 50° to 100°C) than electrosurgical device.

The aim of this systematic review is to evaluate the actual role of UAS versus conventional clamp and tie (CT) in total thyroidectomy.

## Methods of metanalysis

### Search methods for identification of studies

We planned to search for published Randomized Controlled Trials (RCTs) and Controlled Clinical Trials (CCTs), without language restrictions, using the following electronic databases:

• Cochrane Central Register of Controlled Trials (January 2010);

• MEDLINE (1966 to January 2010);

• EMBASE (1980 to January 2010);

• Science Citation Index (1981 to January 2010);

• ISI Proceedings (1990 to January 2010);

• Zetoc (searched January 2010);

• CINAHL (1982 to January 2010);

• Clinicaltrials.gov (searched January 2010).

The literature searches were carried out using the following medical subject headings (MeSH) and free text words: ''thyroidectomy''; "Harmonic scalpel", "Ultrasonic dissector". We also checked the reference lists of all studies identified.

### Data Extraction

Two authors (RC, FD) assessed titles or abstracts of 56 all the studies identified by the initial search and excluded clearly non-relevant studies. They obtained the full text of all potentially relevant studies and also those with unclear methodology. These studies were assessed by the two authors as to whether they met the inclusion criteria for this review. One disagreements on inclusion were resolved by discussing and, if necessary, by involving an independent third author (ST).

### Inclusion Criteria

To be included in the analysis, the studies had to compare total thyroidectomy with UAS versus CT.

### Exclusion Criteria

Studies were excluded from the meta-analysis if the outcomes of interest were not reported for the two techniques or there was a considerable overlap between authors, centres or patient cohorts evaluated. More extended surgical procedure adding central neck dissection (level 6 lymphadenectomy) were excluded.

### Outcomes of Interest

The following outcomes were used to compare the total thyroidectomy group with UAS versus CT group:

• Operative duration (minutes).

• Operative blood loss (mL).

• Overall drainage volume (mL) during the first 24 hours.

• Transiet laryngeal nerve palsy (no. of patients).

• Permanent laryngeal nerve palsy (no. of patients).

• Transiet hypocalcaemia (no. of patients).

• Permanent hypocalcaemia (no. of patients).

### Methodological quality

RC, DF and ST assessed the methodological quality of each trial independently.

### Measures of treatment effect

Data were analyzed for odds ratio (OR) in the case of dichotomous variables, and weighted mean difference (WMD) for continuous variables. Ninety five % confidence intervals (95% CI) were calculated for these measures of effect. Intention-to-treat analyses were performed extracting the number of patients originally allocated to each treatment group irrespective of compliance. The Mantel-Haenszel method was used for the meta-analysis. Results were presented on a forest plot graph.

### Assessment of heterogeneity

The Chi2 test was employed for heterogeneity assessment. The outcomes were measured with continuous scales, while data of treatment effects were analysed with mean difference. If different trials used different scales, we standardized and combined the results (i.e. standardized mean difference).

### Statistical Analysis

The data analysis was performed using the meta-analysis software Review Manager (RevMan) v 5.0.17 (Copenhagen: The Nordic Cochrane Centre, The Cochrane Collaboration, 2008).

## Results for the Meta-Analysis

### Eligible Studies

There are currently 8 RCT on this issue to compare thyroidectomy with UAS (First generation "Harmonic scalpel"; Johnson & Johnson^®^) versus CT [4-11], a study is excluded for the impossibility to distinguish lobectomy from total thyroidectomy [11].

From the analysis of 7 studies it was possible to confront 608 cases divided into two groups [4-10] (Table 1): 303 undergoing to total thyroidectomy with UAS versus 305 that were treated with CT.

**Table 1:** RCT included

Authors, year	No of patients		Age, Mean (SD), y		Pathological diagnosis of Lesions, B/M		Operative Duration, Mean (SD), min		Operative Blood Loss Mean (SD), ml		Length of Hospital Stay, Mean (SD), d		No. of Patients with Nerve plasy				No. of Patients with Hypocalcemia			
													
												Transiet		Permanent		Transiet		Permanent		
	
	HS	CT	HS	CT	HS	CT	HS	CT	HS	CT	HS	CT	HS	CT	HS	CT	HS	CT	HS	CT
Defechereux 2003	17	17	48.1 (13.9)	52.1 (11.1)	17/0	17/0	70.7 (18.3)	96.5 (28.9)	74.5 (50.9)	134.6 (108.4)	2.87 (0.35)	3.0 (0.59)	0	0	0	0	1	4	0	0

Ortega 2004	57	57	53.5	52.5	57/0	57/0	86 (20)	101 (16)	---	---	1.07	1.15	3	2	0	0	5	6	0	0

Cordòn 2005	29	37	---	---	0/7	2/10	96 (23)	21 (34)	35 (27)	54 (51)	ND	ND	1	0	0	0	3	9	0	0

Miccoli 2006	50	50	47	44	47/3	47/3	40 (6.8)	46.7 (10.8)	---	---	ND	ND	5	16	0	0	0	0	0	0

Hallgrimsson 2008	27	24	42	34	27/0	24/0	121	172	69	79	ND	ND	4	1	0	0	8	11	0	1

Lombardi 2008	100	100	49.5 (14.2)	52.5 (23.4)	73/27	61/39	53.1 (20.7)	75.2 (23.5)	---	---	4.3 (1.5)	4.3 (1.3)	2	1	0	0	28	29	0	0

Papavramidis 2009	45	45	48.78 (14.70)	49.39 (11.59)	---	---	76.67 (22.88)	101.74 (20.76)	---	---	2.61 (0.18)	3.24 (0.21)	0	0	0	0	ND	ND	ND	ND

### Results

**Figure 1:**
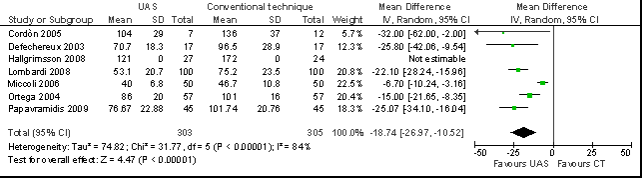
**Meta-analysis of operative duration (min) in total thyroidectomy with ultrasonic dissector of the first generation versus conventional clamp and tie**.

The operative time (WMD = -18.74 minutes; 95% CI -26.97 to -10.52 minutes) was statistically relevant lower in the total thyroidectomy with UAS group (*p *= 0.00001) (Figure 1).

**Figure 2:**
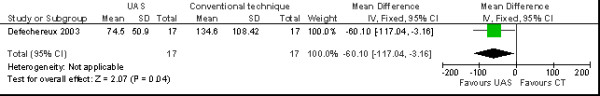
**Meta-analysis of operative blood loss (ml) in total thyroidectomy with ultrasonic dissector of the first generation versus conventional clamp and tie**.

The intraoperative blood loss is only mentioned in one included trial (WMD = -60.10 mL; 95% CI -117.04 to 3.16 mL) was lower in the total thyroidectomy with UAS group (*p *= 0.04) (Figure 2).

**Figure 3:**
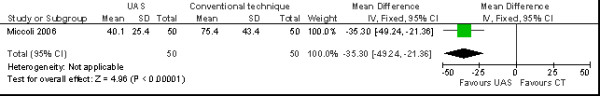
**Meta-analysis of overall drainage volume (mL) in total thyroidectomy with ultrasonic dissector of the first generation versus conventional clamp and tie**.

The overall drainage volume in one included trial (WMD = -35.30 mL; 95% CI -49.24 to 21.36 mL) was significant lower in the total thyroidectomy with UAS group (*p *= 0.00001) (Figure 3).

**Figure 4:**
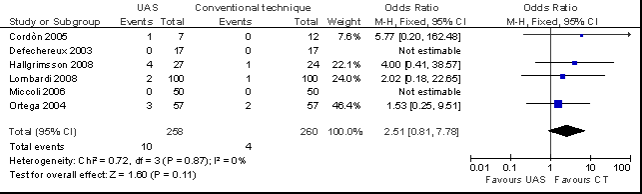
**Meta-analysis of incidence of transiet nerve plasy (n. patients) in total thyroidectomy with ultrasonic dissector of the first generation versus conventional clamp and tie**.

**Figure 5:**
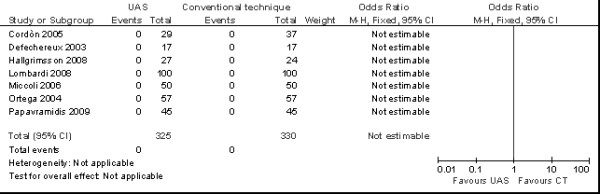
**Meta-analysis of incidence of permanent nerve plasy (n. patients) in total thyroidectomy with ultrasonic dissector of the first generation versus conventional clamp and tie**.

The incidence of transient laryngeal nerve palsy (OR = 2.51 no. of patients; 95% CI 0.81 to 7.78 no. of patients) was fewer in the total thyroidectomy with UAS group (*p *= 0.11) (Figure 4), but not confer any statistically significant advantage over CT; the incidence of permanent laryngeal nerve palsy was similar in the two groups (Figure 5).

**Figure 6:**
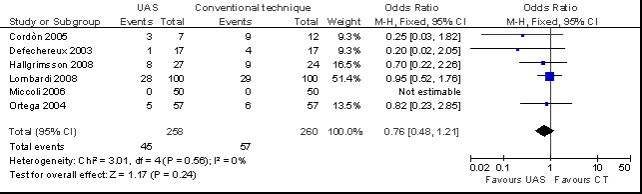
**Meta-analysis of incidence of transient hypocalcaemia (n. patients) in total thyroidectomy with ultrasonic dissector of the first generation versus conventional clamp and tie**.

**Figure 7:**
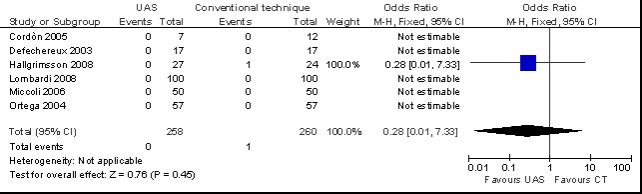
**Meta-analysis of incidence of permanent hypocalcaemia (n. patients) in total thyroidectomy with ultrasonic dissector of the first generation versus conventional clamp and tie**.

There aren't relevant differences in the incidence of transient (OR = 0.76 no. of patients; 95% CI 0.48 to 1.21 no. of patients) (*p *= 0.24) (Figure 6) and permanent hypocalcaemia (OR = 0.28 no. of patients; 95% CI 0.01 to 7.33 no. of patients) (*p *= 0.45) was similar in the two groups (Figure 7).

## Discussion

The ultrasonic dissector, although more costly, has gained wide acceptance because it may reduce intraoperative bleeding and operative duration. Reported benefits, however, were based on some RCTs and CCTs and conclusive evidence supporting either technique is lacking.

We conducted a systematic review and meta-analysis to compare the surgical effectiveness of ultrasonic vs clamp and tie in total thyroidectomy in patients with thyroid benign and/or malignant pathologies.

From our systematic review and meta-analysis the main advantages of UAS are shorter operative duration (*p *= 0,00001), lower intraoperative blood loss (*p *= 0,04) and lower overall drainage volume (*p *= 0,00001).

The significant advantage of this device is the simultaneously coagulating/dissecting functions, and subsequent timing reduction necessary for conventional clamp-and-tie technique. This advantage was evidenced by Yao and coll. in their systematic review and meta-analysis on usage of Ligasure in total thyroidectomy [12] (WMD = -18.74 minutes in patients treated with con Ultrasonic dissector versus-20.32 minutes in patients treated with LigaSure). The reduction of operative time has the advantage to reduce significantly the costs of utilization of operating room.

The cut and coagulation functions permit also to reduce lymphorrea depending on ligations and sections, so the opportunity of anticipated demission and subsequent reduction of costs of permanence in the Hospital.

The utilization of Ultracision permits a more accurate dissection and a statistically significant reduction of intraoperative blood loss compared to the patients treated with conventional vessel ligation in thyroidectomy, no presence of advantage in patients treated with Ligasure [12] (WMD = -60.10 mL in patients treated with Ultrasonic dissectors, *p *= 0.02, versus -25.13 mL in patients treated with LigaSure, *p *= 0.26).

The incidence of post-operative complications is similar in two groups (transient laryngeal nerve palsy: *p *= 0.11; permanent laryngeal nerve palsy: not estimable; transient hypocalcaemia: *p *= 0.24; permanent hypocalcaemia: *p *= 0.45). In the patients treated with Ultrasonic dissector we have only a little reduction of complications; that can be attributed to the technical characteristics of UAS that reduce lateral thermal injury, approximately half that caused by bipolar systems.

The hypocalcaemia is subsequent to parathyroid glands damage during thyroidectomy. The transient hypocalcaemia appears in 10% of total thyroidectomies, permanent in 1% [13-19]. The transient hypocalcaemia could be also severe and needs to be treated with iv or oral therapy. The permanent hypocalcaemia requests a long-life treatment with calcium supplements and vitamin D analogues. The long-life treatment with supplements could have some inconveniences like osteomalacia. The incidence of parathyroid glands damage could be reduced only performing a precise surgical techniques to preserve blood supply.

In the most cases, laryngeal nerve palsy is not frequent and transient (10% after total thyroidectomy) and can be resolved spontaneously maximum in one month[13-19]; permanent paralysis is very rare (1%) and need a complex treatment (vocal cord injection or laryngoplasty) [13-19].

The prevention of those lesions is of high importance and can be obtained only with an accurate dissection.

The experience and choice of surgical techniques represent the unique cause of hypocalcaemia and laryngeal nerve palsy, Kocher evidenced this problem: "Since we have adhered strictly to this procedure, the hoarseness, formerly so frequently observed after operation, has now become exceptional". Recently a new UAS handpiece was commercialized (Harmonic Focus) with a tip smaller than 5 mm. These smaller tips should permit more precise and accurate dissection with a subsequent reduction of post operative complications.

Actually, it was shown from this meta-analysis of the seven randomized clinical trials (RCT) a relevant advantage only in terms of cost-effectiveness (reduction of operating room utilization and recovering) in patients treated with UAS (first generation "Harmonic scalpel"; Johnson & Johnson^®^), it is subsequent to statistically significant reduction of operation duration (*p *= 0.00001), intraoperative blood loss (*p *= 0.04) and of overall drainage volume during the first 24 hours (*p *= 0.00001). Although the analysis showed that the patients who were treated with Ultrasonic dissector presented more favourable results in incidence of post-operative complications (transient laryngeal nerve palsy: *p *= 0.11; permanent laryngeal nerve palsy: not estimable; transient hypocalcaemia: *p *= 0.24; permanent hypocalcaemia: *p *= 0.45), these data didn't present statistical relevance.

The experience of surgeon is the only significant factor of appearance of complications, utilization of Ultrasonic dissector can only facilitate surgical procedure, but can not substitute the experience of surgeon.

It's necessary to do new and more enlarged RCT with new UAS Focus, this should permit a more correct evaluation of advantages of UAS in reduction of post operative complications.

## Competing interests

The authors declare that they have no competing interests.

## Authors' contributions

RC, FD, ST: Literature search and identification of trials, writing the text of review. AS, GR, DV: Evaluation of methodological quality of trials, data collection. RL, DG, AS: Literature search and identification of trials. FS, AB, AN: Revision of the review.

All authors read and approved the final manuscript.
